# Benign and malignant prostate neoplasms show different spatial organization of collagen

**DOI:** 10.3325/cmj.2023.64.413

**Published:** 2023-12

**Authors:** Taras Zadvornyi, Nataliia Lukianova, Oleksandr Mushii, Anna Pavlova, Olena Voronina, Vasyl Chekhun

**Affiliations:** Department of Monitoring Tumor Process and Therapy Design, R.E. Kavetsky Institute of Experimental Pathology, Oncology and Radiobiology of the National Academy of Sciences of Ukraine, Kyiv, Ukraine

## Abstract

**Aim:**

To compare the indicators of the spatial organization of collagen and its regulating factors between benign and malignant prostate neoplasms.

**Methods:**

The study involved tumor tissue samples from 40 patients with stage II-III prostate cancer (PCa) and 20 patients with benign prostatic hyperplasia (BPH). The localization of collagen was determined with a Masson trichrome stain. To establish quantitative indicators of the spatial organization of collagen, morphometric studies were carried out with the CurveAlign and ImageJ programs.

**Results:**

PCa tissue had two times lower collagen density (*P* < 0.0001) and 1.3 times lower levels of collagen alignment (*P* = 0.018) compared with BPH tissue. In PCa tissue, collagen fibers were shorter (by 24.2%; *P* < 0.001) and thicker (by 15.5%; *P* < 0.001). PCa tissue samples showed significantly higher levels of metalloproteinase (MMP)-2 (by 2.4 times; *P* = 0.001), MMP-8 (by 2.3 times; *P* = 0.007), and MMP-13 (by 1.9 times; *P* = 0.004).

**Conclusions:**

Collagen matrix spatial organization features, as well as its regulatory factors, could be potential biomarkers of malignant prostate neoplasms.

Disruption of the extracellular matrix (ECM) plays an important role in the development and progression of many malignant neoplasms. The composition and structure of the ECM are unique to each tissue and vary according to its function. The interaction of tumor cells with the ECM affects a number of biological processes, such as proliferation, differentiation, migration, and metabolism (1-3). Normally, the ECM is required to maintain cellular architecture and polarity, which is disrupted during neoplastic growth. During carcinogenesis, the ECM is actively reconstructed, and becomes more structured and rigid due to a high content of collagen. The ECM is remodeled by a coordinated action of many proteolytic systems and cells of the tumor microenvironment, such as fibroblasts or macrophages (4). As a result of remodeling, changes in the composition and structure of the ECM contribute to the abnormal behavior of stromal cells, in particular fibroblasts, and immune and endothelial cells (1). This is why the features of ECM are currently considered as prognostic and diagnostic markers, and the matrix itself as a new therapeutic target (5,6).

The spatial organization of collagen, which is the main structural protein of the ECM (7), is an important modulator of the growth and progression of malignant neoplasms (8,9). Changes in the representation and organization of collagen fibers contribute to the formation of tumor microenvironment, influencing the migration and polarization of stromal cells (10,11). High density and alignment degree of collagen fibers are associated with the prognosis of breast cancer, pancreatic cancer, gastric cancer, and oral squamous cell carcinoma (12-15). In addition, a high content of collagen in the tumor microenvironment may stimulate metastasis (16).

Collagen degradation that occurs during stroma remodeling is maintained by various types of proteases, particularly matrix metalloproteinases (MMPs) or matrixins. MMPs are capable of cleaving collagen, initiating collagenolysis, and making collagen sensitive to degradation by other, less specific proteinases (17). Along with this, MMPs affect the morphological characteristics (width and length) of collagen fibers (18).

Prostate cancer (PCa) is a highly heterogeneous disease both in terms of morphological structure and molecular profile, and its clinical course, and this heterogeneity causes difficulty in diagnosis and therapy (19). At the same time, in clinical practice today, there is a paucity of molecular tumor markers that would allow physicians to accurately diagnose a malignant prostate neoplasm or to confirm the primary diagnosis established after the testing of serum prostate-specific antigen or after a fine needle biopsy. The elements of the tumor microenvironment attract more and more attention from scientists and clinicians, but experimentally confirmed data characterizing changes in the stroma of the tumor center are scarce. Given the role of collagen in tumor progression, the study aimed to assess the quantitative indicators of the spatial organization of collagen in PCa and benign prostatic hyperplasia (BPH) tissue, as well as the factors influencing its regulation.

## MATERIALS AND METHODS

### Study design

The study was carried out in the Department of Tumor Process Monitoring and Therapy Design of the R.E. Kavetsky Institute of Experimental Pathology, Oncology and Radiobiology of the National Academy of Sciences of Ukraine. It was approved by the Bioethics Committee of R.E. Kavetsky Institute of Experimental Pathology, Oncology and Radiobiology of the National Academy of Sciences of Ukraine (Protocol #4 dated 10/16/22) and complied with the Declaration of Helsinki and guidelines for good clinical practice. All patients gave informed consent for the use of clinical data for scientific purposes. The study was conducted on biopsy samples of 40 patients with stage II-III PCa and 20 patients with BPH who were treated from 2015-2021 at the National Cancer Institute of the Ministry of Health of Ukraine. The localization of collagen in the tissues of prostate neoplasms was determined with Masson trichrome staining. To establish quantitative indicators of the spatial organization of collagen, morphometric studies were carried out with the CurveAlign v. 4.0. beta (Laboratory for Optical and Computational Instrumentation, University of Wisconsin, Madison, WI, USA) and ImageJ v. 1.42 on photomicrographs obtained with an AxioCam ICc5 camera (Carl Zeiss, Oberkochen, Germany) and the digital microscopy complex - AxioScope A1 (Carl Zeiss). The expression of MMPs in prostate tissues was determined immunohistochemically.

### Clinical and pathological characteristics of patients

The inclusion criterion was the absence of neoadjuvant hormone therapy at the time of diagnosis and surgical intervention. The main inclusion criterion for BPH samples was the absence of PCa foci observed at a 12-point fine-needle biopsy.

The average age of patients with PCa was 63.2 ± 6.7 (range 50-77) years, and that of patients with BPH was 69.1 ± 5.3 (range 59-78) years. The clinical characteristics of 40 PCa patients are shown in Table 1.

**Table 1:** Clinical characteristics of patients with prostate cancer

Index	Number of patients	
	n	%
Total number of patients	40	100
**Age (years)**		
average	63.2 ± 6.7	
range	50-77	
**TNM stage**		
T2	22	55.0
T3	18	45.0
**Gleason score**		
<7	15	37.5
≥7	25	62.5
**Prostate-specific antigen level**		
<10 ng/mL	13	32.5
≥10 ng/mL	27	63.5

The diagnosis of PCa/BPH was verified by examining histological preparations after transrectal multifocal biopsy of the prostate gland under ultrasound control. The tumor stage was determined based on the TNM Classification (7th edition, 2009), according to the consensus of the International Society of Urological Pathology in 2014 (20) and current standards of diagnosis and treatment of the European Association of Urology, National Comprehensive Cancer Network, and European Society for Medical Oncology.

### Histochemical methods

The prostate tumor tissue (biopsy) was fixed in 10% neutral buffered formalin, and serial 5 μm-thick sections were embedded in paraffin blocks. The sections were deparaffinized in xylene and rinsed in ethanol. In order to study the structural components of the tissues, the sections were stained with hematoxylin and eosin according to standard pathohistological procedures.

**Figure 1:**
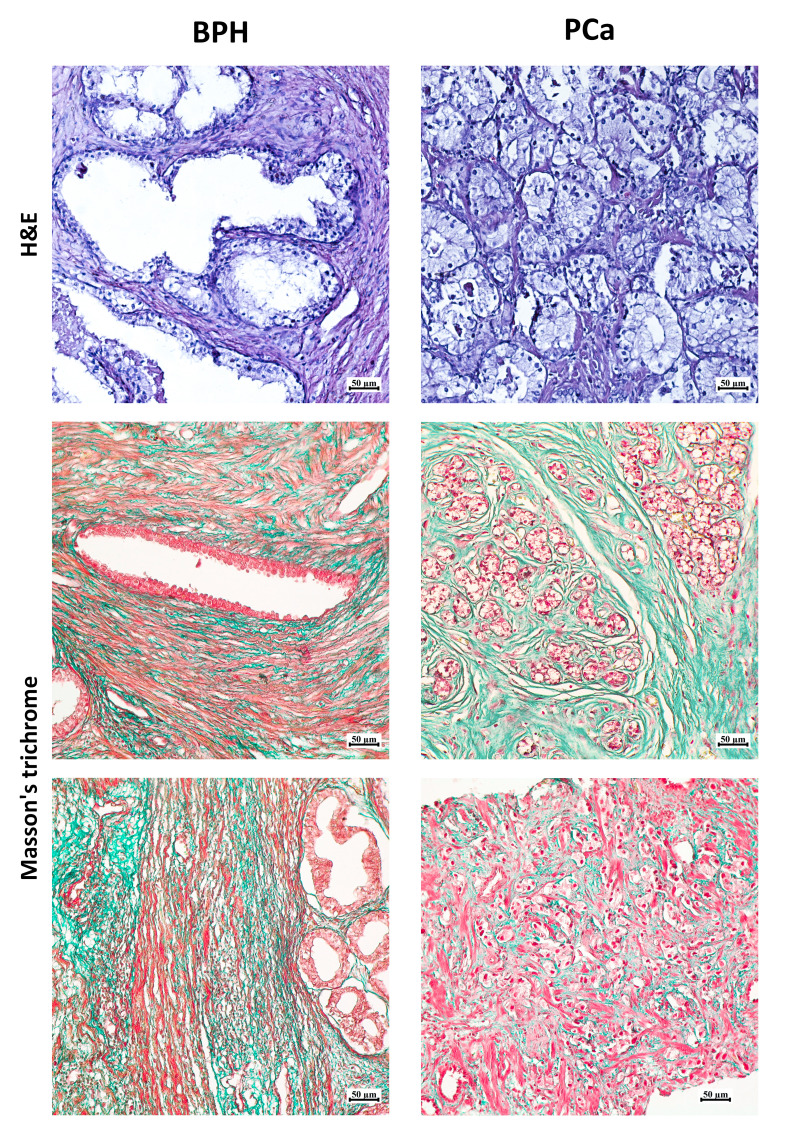
Histological features of malignant (PCa) and benign (BPH) prostate neoplasms. Staining with hematoxylin and eosin (H&E) and Masson’s trichrome. Magnification ×260.

The localization of collagen in the tissues of benign and malignant prostate tumors was identified by Masson trichrome stain. In brief, serial sections of tumor tissue were stained with Masson Trichrome dyes (DiaPath, Martinengo, Italy) according to the manufacturer's recommendations. When Masson's trichrome method was used, muscle fibers, fibrin, acidophilic granules, and cytoplasm were stained red; collagen and basement membrane were stained blue-green; nuclei were stained black-blue; and erythrocytes were stained yellow (Figure 1).

### Morphometric analysis

Quantitative indicators of the organization of collagen fibers in malignant and benign prostate neoplasms were morphometrically studied according to the algorithm by Lukianova et al (21) on microphotographs obtained with a digital microscopy complex - AxioScope A1 and AxioCam ICc5 camera. The image resolution was 2452 × 2056 pixels. The alignment index of collagen fibers was determined with the CT module of CurveAlign v. 4.0. beta (Laboratory for Optical and Computational Instrumentation, University of Wisconsin, Madison, WI, USA) on primary (unedited) photographs of tumor tissue stained by Masson trichrome. When working with images of tissue stained with eosin and hematoxylin, the fractionation coefficient was 0.02.

The length, width, straightness, and orientation of collagen fibers in the neoplastic tissues were measured with the CT-FIRE module of the CurveAlign program, 4.0. beta on preprocessed photomicrographs of tumor tissue stained with Masson trichrome. In particular, with the help of the Adobe Photoshop CC graphic editor (Adobe Systems Inc., San Jose, CA, USA), tumor tissue components were segregated by color, and monochrome images of the collagen network were created and used for further research. When working with images of tissue samples, the fractionation coefficient was 0.001.

The density of the collagen matrix in the tissue was calculated as the ratio of the number of collagen fibers, obtained with the CT-FIRE module, to their area, which was measured with the ImageJ program, v. 1.42 (*https://imagej.nih.gov/ij/).*

When analyzing the quantitative characteristics of the spatial organization of collagen, the parameters of about 1 × 10^6^ collagen fibers in PCa tissue and about 3 × 10^5^ collagen fibers in BPH tissue were studied. The average number of collagen fibers in one image of PCa tissue was ~ 2150 fibers, and in an image of BPH tissue ~ 1500 fibers.

### Immunohistochemical analysis

The expression of MMPs in the tissue of prostate neoplasms was measured on 5 μm-thick paraffin sections. Antigens were unmasked in EDTA buffer, pH = 8.0 (Diagnostic BioSystems, Pleasanton, CA, USA) in a WB-4MS water bath (Biosan, Riga, Latvia) for 20 min at 96 °C. Monoclonal antibodies specific to MMP-1 (clone 6A5; Thermo Scientific, Waltham, MA, USA), MMP-2 (clone 101; Thermo Scientific), MMP-9 (clone EP1254; Abcam, Cambridge, UK), MMP-13 (clone VIIIA2; Thermo Scientific), and polyclonal antibodies specific to MMP-8 (ab53017; Abcam) were used as primary antibodies. To visualize the results of the reaction, a set of Mouse/Rabbit PolyVue Plus HRP/DAB Detection System reagents was used (Diagnostic BioSystems, Pleasanton, CA, USA) as per the manufacturer's recommendations. Sections were stained with Meyer's hematoxylin (Thermo Scientific Richard-Allan, Kalamazoo, MI, USA).

Immunopositive cells were counted under a Primo Star light microscope (Carl Zeiss, Oberkochen, Germany) at a magnification of ×400. The H-Score method was used to quantify the expression of the studied molecules:

S = 0 × N_0_ (%) + 1 × N_1_ (%) + 2 × N_2_ (%) + 3 × N_3_ (%),

where S is the “H-Score” index; N_0_ – the number of cells with no expression of the studied marker; N_1_, N_2_ and N_3_ – the number of cells with low, medium, and high expression, respectively. The final result of the calculation was expressed in points: 1 -100 points – low level of expression; 101-200 points – medium level of expression; and 201-300 - high level of expression (22).

### Statistical analysis

The significance of differences was assessed with the Mann-Whitney U-test. Quartile diagrams (“boxplot”) were used to graphically present the results of the study. *P* < 0.05 was considered significant. Statistical analysis was performed with GraphPad Prism v.8.0 (GraphPad Software Inc., La Jolla, CA, USA).

## RESULTS

### The topology of collagen fibers in the tissue of benign and malignant prostate neoplasms

The analysis of cytomorphological features showed a large amount of connective tissue stroma between the small glands in the BPH tissues. As a result, the acini sometimes had a flattened, irregular shape (Figure 1). Another characteristic feature of BPH was the overgrowth of fibrous connective tissue containing an increased number of fibroblasts and myofibroblasts. In PCa tissues, we observed varying preservation of glandular structures, and their abnormal structure; an absence of a basal layer of cells; loss of polarity; large nuclei; and the presence of mitosis. The basement membrane had a non-uniform thickness and a weak connection with the epitheliocytes.

When the topology of collagen fibers in BPH tissues was studied, uniform red and green staining was recorded, which indicated the same volume of collagen and smooth muscle fibers in the stroma. On the other hand, PCa tissues were characterized by a significantly decreased content of smooth muscle elements and a significant increase in the collagen component. Collagen fibers in the BPH tissues were usually located around vessels and were characterized by different thickness and chaotic organization. PCa tissues showed a disrupted structural organization of the collagen fibers (looseness and decreased density in the hypertrophied stroma), which were most often localized around the ducts and acini, in the basal membranes of epithelial structures, and around blood vessels (Figure 1).

### Morphometric study of the parameters of the spatial organization of collagen in the tissue of benign and malignant prostate neoplasms

**Figure 2:**
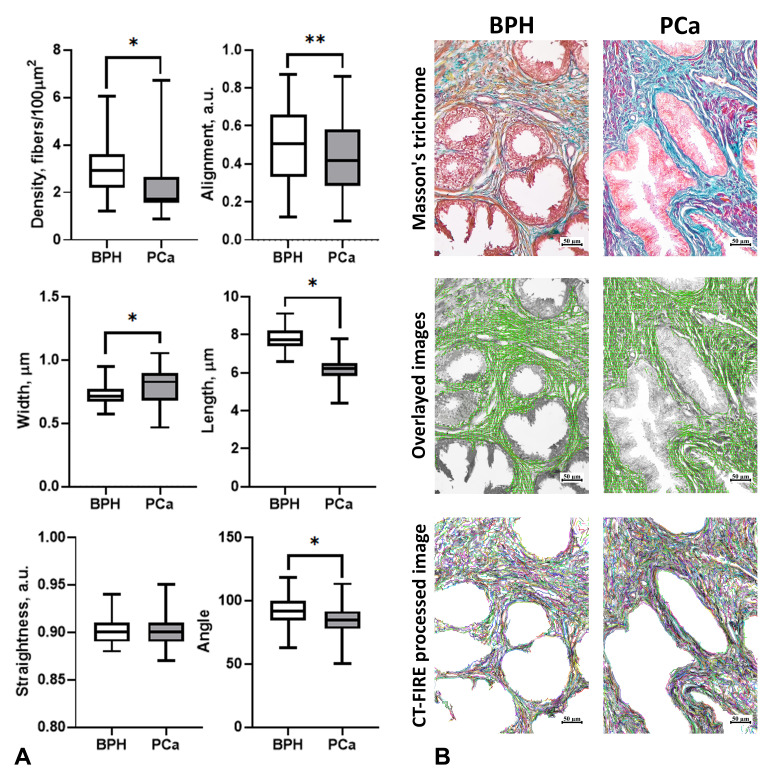
(**A**) Indicators of the spatial organization of collagen in benign (BPH) and malignant prostate neoplasms (PCa). (**B**) Representative images of the morphology of collagen fibers in BPH and PCa stained with Masson trichrome and analyzed with the CT-FIRE module of the CurveAlign program. Magnification ×260. *P < 0.0001; **P < 0.01.

The morphometric study of the quantitative characteristics of collagen fibers confirmed the data obtained in the pathological analysis (Figure 2). In particular, PCa tissues were characterized by two times (*P* < 0.001) lower collagen density and 1.3 times (*P* = 0.018) lower indicators of collagen alignment compared with BPH tissues.

Collagen fibers in PCa tissues were significantly shorter (by 24.2%; *P* < 0.001) and wider (by 15.5%; *P* < 0.001). The index of the orientation of collagen fibers in PCa tissues was significantly lower compared with that in BPH tissues (*P* < 0.001). There was no significant difference in the coefficients characterizing the straightness of collagen fibers.

### The expression of MMPs in the tissues of benign and malignant prostate neoplasms

**Figure 3:**
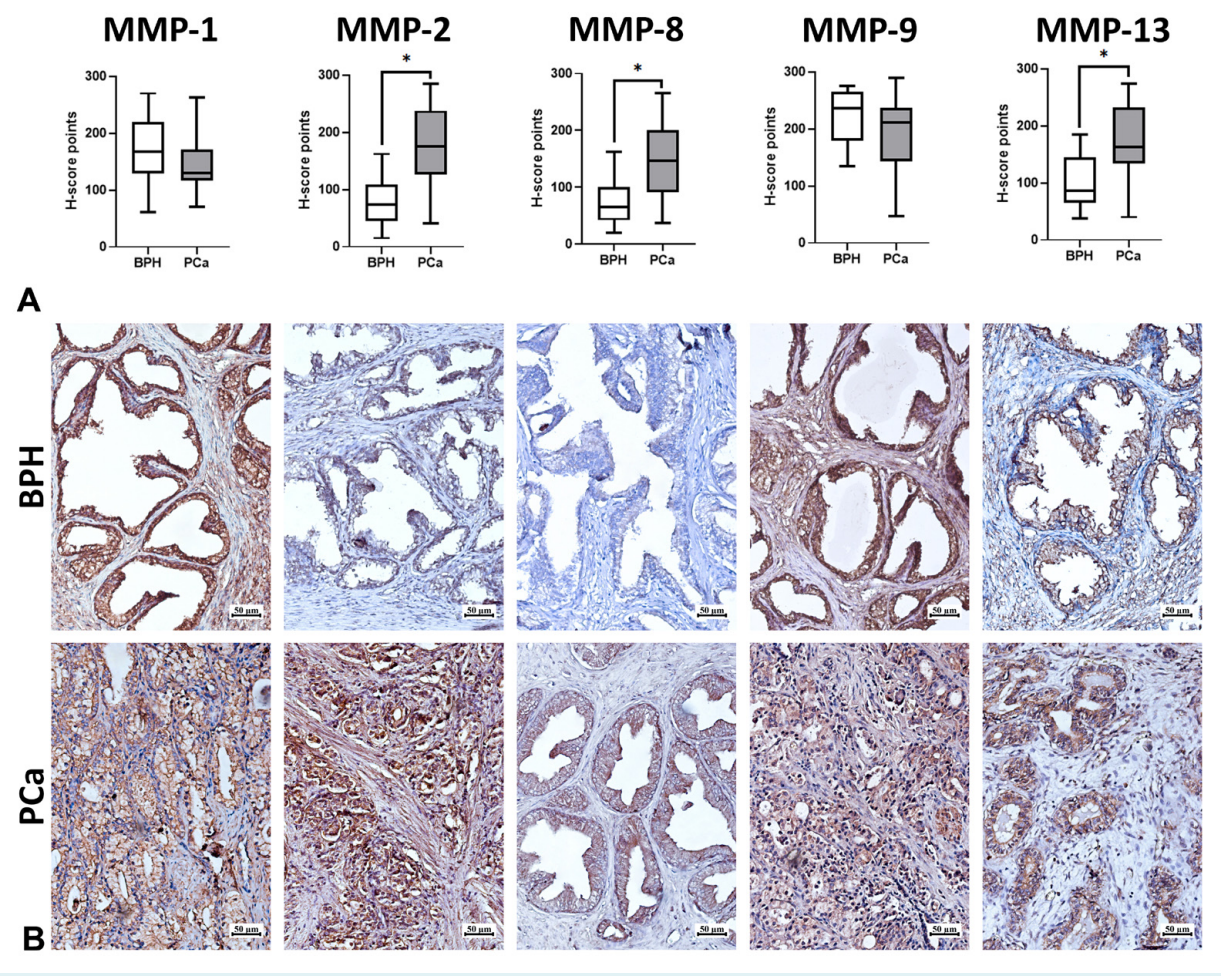
Expression of metalloproteinases (MMPs) in benign and malignant prostate neoplasms. (**A**) Quantitative indicators of MMPs expression; (**B**) Representative photomicrographs of the expression of MMPs in prostate cancer (PCa) and benign prostatic hyperplasia (BPH). Immunohistochemistry, chromogen 3-diaminobenzidine tetrachloride. Staining with Meyer's hematoxylin. Magnification ×260. *P < 0.01.

PCa tissues had significantly higher levels of MMP-2 (2.4 times; *P* = 0.001), MMP-8 (2.3 times; *P* = 0.007), and MMP-13 (1.9 times; *P* = 0.004) compared with BPH tissue (Figure 3). There was no significant difference between the groups in the expression levels of type I collagenase, MMP-1, or MMP-9.

## DISCUSSION

In this study, we identified decreased ECM density and length and an increased thickness of collagen fibers in PCa samples, which occurred as a result of active remodeling processes controlled by MMPs, in particular MMP-2, MMP-8, and MMP-13.

During the last decades, research on the emergence and progression of malignant neoplasms has shifted its focus from the malignant-transformed cells to their microenvironment. At the same time, the attention has mainly been on the cellular component of the microenvironment, while the role and changes of individual stromal elements, in particular the ECM, remain poorly characterized. The stroma performs a supporting function, serving as a framework for tissues and organs. It is also a key factor in maintaining the stability of the tumor microenvironment, providing nutrients and transmitting signals between the cells. Therefore, determining the role of each stromal element in carcinogenesis could be useful in cancer diagnosis and treatment (23).

Collagen is the most abundant component of the ECM, but its role in the processes of malignant growth is not fully understood. In view of this, our study found that the structure of collagen in PCa tissue was significantly different compared with that in BPH tissues. In particular, PCa tissue was characterized by a lower density of the collagen matrix, as well as a decreased length and an increased thickness of collagen fibers. The features of the structural organization of collagen in malignantly transformed tissues can stimulate tumor growth and invasion, and be associated with high metastatic activity of neoplasms. Collagen fibers can act as a “highway” for neoplastic cells (8). As a result of stroma remodeling, which occurs during the progression of malignant neoplasms, collagen fibers in the tumor tissue are located perpendicularly, thus providing channels for the penetration of malignant cells through the basement membrane (24). This is confirmed by studies in an *in vitro* system, which showed that an increased thickness and length of collagen fibers were associated with an increased invasiveness of breast cancer cells (25). Furthermore, linearized collagen fibers increased ECM stiffness, which, in turn, also contributed to increased tumor malignancy (25,26). However, Bodelon et al (27) reported that decreased length, straightness, width, and density of collagen fibers, as well as increased fiber alignment, were associated with increased local damage to normal tissue by breast tumors.

The differences in the morphological characteristics of collagen and its spatial organization established in this study indicate a high remodeling activity of PCa stroma. The key role in these processes is played by MMPs, which are also involved in the regulation of important biological processes, such as cell growth, differentiation, migration, and angiogenesis, and also indirectly affect the chemotaxis of immune cells (28). During the cleavage of collagen by MMPs, its fragments, matrikines, can stimulate the recruitment of tumor-associated macrophages, a process contributing to the emergence and progression of prostate neoplasms (8,28).

Some MMPs are capable of catalytically splitting and degrading collagens of the fibrillar type, thereby contributing to the restructuring of the fibrous component of the stroma of prostate neoplasms (17). According to our data, PCa tissues were characterized by high and medium expression levels of all MMPs, a finding indicating active processes of tumor stroma remodeling. The significant increase in the expression of MMP-2, MMP-8, and MMP-13 in PCa tissues observed in our study is consistent with the existing literature data on the organization of the fibrous matrix of the stroma (29).

The results of this study should be considered in light of some limitations. First, the sample size of both BPH and PCa was small. Second, more sensitive and specific methods, such as second-harmonic generation microscopy or histochemical staining of Picro-Sirius Red in combination with polarization microscopy, can be used to identify collagen fibers in the stroma of PCa. Third, we used biopsy material, which cannot fully reflect the entire picture of pathological changes in the collagen framework in the prostate gland in the presence of benign and malignant neoplasms. The use of postoperative material, and ideally full-size sections of the affected prostate gland and their images obtained with a slide scanner could improve the quality of the analysis.

Taking into account the existing data on the relationship between the expression of individual MMPs and the clinical and pathological characteristics of patients with PCa (29,30), there is a need for further studies on the quantitative and qualitative parameters of collagen fibers. These parameters can be used as biomarkers associated with the aggressiveness of the tumor process.
